# Nanoencapsulation of *Artemisia annua* Essential Oil in Chitosan-Lipid Carriers Enhances Stability, Larvicidal, Antifungal, and Anticancer Efficacy

**DOI:** 10.3390/pharmaceutics18070804

**Published:** 2026-06-29

**Authors:** Ghassab M. Al-Mazaideh, Mohammed Alshammari, Bader Alsuwayt, Abdulkareem A. Alanezi, Nimer Fehaid Alsabeelah, Afaf F. Almuqati, Meshal Alotaibi, Shatha Alzahrani, Turki Hamdan Alsayyali, Haya Ayyal Salman, Abdulrahman Fahad Nagi Almutairi, Mohammed Helmy Faris Shalayel

**Affiliations:** 1 Department of Pharmaceutical Chemistry, College of Pharmacy, University of Hafr Al Batin, Hafr Al Batin 39524, Saudi Arabia; aalmaqati@uhb.edu.sa; 2 Department of Pharmacy Practice, College of Pharmacy, University of Hafr Al Batin, Hafr Al Batin 39524, Saudi Arabia; moalshammari@uhb.edu.sa (M.A.); balsuwayt@uhb.edu.sa (B.A.); dr.nalsabeelah@uhb.edu.sa (N.F.A.); motaibi@uhb.edu.sa (M.A.); mhfshalayel@uhb.edu.sa (M.H.F.S.); 3 Department of Pharmaceutics, College of Pharmacy, University of Hafr Al Batin, Hafr Al Batin 39524, Saudi Arabia; aalanezi@uhb.edu.sa; 4 Department of Clinical Laboratory Sciences, College of Applied Medical Sciences, Taif University, Taif 21944, Saudi Arabia; shalzahr@tu.edu.sa; 5 College of Pharmacy, University of Hafr Al Batin, Hafr Al Batin 39524, Saudi Arabia; turki.pharmd1@gmail.com; 6 School of Biological Sciences, Universiti Sains Malaysia, 1112, Persiaran Sains, Gelugor 11800, Pulau Pinang, Malaysia; hayaayyalsalman@stdent.usm.my; 7 Deanship of Research and Innovation, University of Hafr Al Batin, Hafr Al Batin 39524, Saudi Arabia; afalmutairi@uhb.edu.sa

**Keywords:** *Artemisia annua* essential oil, chitosan-coated nanostructured lipid carriers, nanoencapsulation, larvicidal activity, antifungal activity, apoptosis

## Abstract

**Background/Objectives:** *Artemisia annua* essential oil (EO) possesses diverse biological activities; however, its practical application is limited by volatility, instability, and poor bioavailability. This study aimed to develop chitosan-coated nanostructured lipid carriers (CH-NLCs) for efficient encapsulation and delivery of *A. annua* EO and to evaluate their physicochemical characteristics and biological performance. **Methods:** The nanoformulation exhibited favorable physicochemical properties, including a high encapsulation efficiency (85.97 ± 1.30%) and a strongly positive surface charge (approximately +45 mV), indicating good colloidal stability. Structural analyses by SEM, FTIR, and XRD confirmed successful encapsulation of the EO within the nanocarrier matrix. **Results:** The CH-NLC formulation significantly enhanced larvicidal activity against Aedes aegypti larvae, reducing the LC_50_ value from 213 ppm for the free EO to 142 ppm. Enhanced antifungal activity was also observed, with 47–56% greater inhibition against Malassezia furfur, Trichophyton mentagrophytes, and *Candida albicans* compared with the free EO. Furthermore, CH-NLC demonstrated improved cytotoxic activity against skin cancer cell lines, achieving IC_50_ values of 21.4 ± 1.7 µg/mL and 30.1 ± 1.6 µg/mL against A431 and A375 cells, respectively, while maintaining lower toxicity toward normal HaCaT keratinocytes. Mechanistic investigations revealed enhanced apoptosis and an approximately 3-fold increase in intracellular reactive oxygen species (ROS) levels in treated cancer cells. **Conclusions:** Collectively, these findings indicate that chitosan-coated nanostructured lipid carriers effectively improve the stability and biological efficacy of *A. annua* essential oil and represent a promising platform for future biomedical and biocidal applications.

## 1. Introduction

The rising incidence of vector-borne diseases and the growing challenge of antimicrobial resistance have intensified the search for effective and sustainable alternatives to conventional chemical agents [1]. Essential oils are well recognized for their anti-inflammatory properties, which are particularly beneficial in pulmonary infections where exaggerated inflammatory responses contribute to tissue damage and disease progression. In parallel, chitosan-based delivery systems can be structurally modified—such as through trimethylation—to enhance pulmonary permeability, mucus interaction, and immunomodulatory capacity, thereby improving their suitability for respiratory drug delivery [2].

In this context, plant-derived essential oils (EOs) have emerged as promising candidates due to their broad-spectrum bioactivity, biodegradability, and generally recognized as safe (GRAS) status [3]. *Artemisia annua* L. (*A. annua*), in particular, has garnered significant scientific interest beyond its well-documented antimalarial properties. Its essential oil is a complex mixture of monoterpenes and sesquiterpenes, such as camphor, artemisia ketone, and 1,8-cineole, which have demonstrated potent insecticidal, antifungal, and cytotoxic activities [4,5].

Despite their potential, the practical application of EOs is often hampered by intrinsic limitations, including high volatility, poor aqueous solubility, chemical instability under environmental factors (light, oxygen, heat), and susceptibility to rapid degradation [6]. These drawbacks lead to a short half-life and diminished bioavailability, necessitating frequent and high-dose applications which are neither economical nor environmentally ideal [7].

Nanoencapsulation strategies offer a powerful solution to overcome these challenges. Among various nanocarriers, nanostructured lipid carriers (NLCs) have gained prominence for their ability to encapsulate lipophilic bioactive compounds, providing enhanced stability, controlled release, and improved bioavailability [8]. Furthermore, surface functionalization of NLCs with biopolymers like chitosan (CH), a natural polysaccharide, can significantly augment their efficacy. Chitosan confers a positive surface charge, which promotes mucoadhesion and enhances interaction with negatively charged biological membranes, such as those of insect cuticles, fungal cell walls, and mammalian cancer cells [9,10]. The resulting chitosan-coated NLCs (CH-NLCs) thus represent a synergistic platform, combining the high payload capacity of lipids with the bioadhesive and permeation-enhancing properties of chitosan.

Recent studies have begun to validate this approach. For instance, nanoencapsulation of *Zataria multiflora* essential oil improved its larvicidal efficacy against Anopheles stephensi [11]. Similarly, chitosan nanoparticles loaded with *Cinnamomum zeylanicum* essential oil demonstrated enhanced antifungal activity against *Candida albicans* (*C. albicans*) compared to the free oil [12]. However, a comprehensive evaluation of a CH-NLC system encapsulating *A. annua* essential oil, correlating its physicochemical properties with multi-faceted biological activities (larvicidal, antifungal, and anticancer), remains largely unexplored.

Despite the growing interest in *A. annua* essential oil and lipid-based nanoformulations, several limitations remain. Previous studies have primarily focused on either the biological activities of the free essential oil or the development of conventional nanoencapsulation systems, often targeting a single biological application. Moreover, limited attention has been given to the use of chitosan-coated nanostructured lipid carriers as multifunctional delivery platforms for *A. annua* essential oil. Chitosan coating may provide additional advantages, including enhanced colloidal stability, improved retention of volatile bioactive constituents, increased interaction with biological membranes, and prolonged biological activity. Therefore, the development of a chitosan-functionalized nanostructured lipid carrier system may represent a promising strategy to improve the efficacy and stability of *A. annua* essential oil for biomedical and biocidal applications.

Despite the growing body of research on *A. annua* essential oil and lipid-based nano-delivery systems, important challenges remain regarding the stability, controlled release, and biological efficacy of essential oil formulations. Furthermore, studies integrating *A. annua* essential oil with chitosan-coated nanostructured lipid carriers (CH-NLCs) and comprehensively evaluating their larvicidal, antifungal, and anticancer activities within a single platform remain limited. The incorporation of chitosan as a surface-coating material is expected to provide additional advantages, including improved colloidal stability, enhanced retention of volatile constituents, increased interaction with biological membranes, and potentially improved biological performance.

Therefore, this study aimed to develop, characterize, and evaluate a novel CH-NLC nanoformulation for the efficient delivery of *A. annua* essential oil. We hypothesized that the CH-NLC system would significantly enhance the oil’s stability, bioavailability, and bioactivity compared with the non-encapsulated essential oil. The specific objectives were to (1) extract and chemically characterize *A. annua* essential oil using GC–MS; (2) fabricate and physicochemically characterize the oil-loaded CH-NLCs, including particle size, zeta potential, crystallinity, and molecular interactions; (3) assess their larvicidal efficacy against *Aedes aegypti* larvae; (4) evaluate their antifungal activity against a panel of pathogenic fungi; and (5) investigate their anticancer effects and underlying mechanisms, including cytotoxicity, apoptosis induction, and intracellular ROS generation in human cancer cell lines. Through this integrated approach, the study seeks to demonstrate the added value of chitosan-functionalized NLCs as a multifunctional delivery platform capable of enhancing the therapeutic and biocidal potential of *A. annua* essential oil for public health and biomedical applications.

## 2. Materials and Methods

### 2.1. Plant Material Collection and Authentication

Aerial parts of *A. annua* were collected before the flowering stage (July 2023) from the experimental farm of the Faculty of Pharmacy, Cairo University, Giza, Egypt (GPS coordinates: 30.0276° N, 31.2101° E). Botanical identification was conducted by Prof. Ahmed Hassan (Professor of Taxonomy, Department of Botany, Faculty of Science, Cairo University) following standard morphological characterization methods described in the European Pharmacopoeia [13]. A voucher specimen (ARC-2023-07-15) was deposited in the Herbarium of the Botany Department, Faculty of Science, Cairo University. The plant material was shade-dried at ambient temperature (25 ± 2 °C) for 10 days and mechanically ground to a coarse powder (2 mm sieve).

### 2.2. Essential Oil Extraction

Volatile oil was extracted by hydrodistillation for 3 h using a Clevenger-type apparatus according to the standard methods described in the European Pharmacopoeia [14,15]. The oil yield was calculated as volume per 100 g of dry plant material (*v*/*w*). The collected essential oil was dehydrated using anhydrous sodium sulfate (Sigma-Aldrich, St. Louis, MO, USA, purity ≥ 99%) and stored in sealed amber vials at 4 °C until further analysis.

### 2.3. Physicochemical Characterization

Organoleptic properties (color and odor) were evaluated under standardized daylight conditions. Refractive index was measured at 20 °C using an Abbe refractometer (ATAGO PAL-RI). Specific gravity was determined at 25 °C according to AOAC Official Method 920.212 [16]. Optical rotation was analyzed using a digital polarimeter (Rudolph Research Analytical Autopol IV, Hackettstown, NJ, USA) at sodium D-line (589 nm). Solubility was tested in various solvents including ethanol (96%), dimethyl sulfoxide, and n-hexane.

### 2.4. Gas Chromatography–Mass Spectrometry (GC-MS) Analysis

Chemical composition analysis was performed using an Agilent 7890B GC system coupled with 5977B MSD (Agilent Technologies, Santa Clara, CA, USA). Separation was achieved on a DB-5MS capillary column (30 m × 0.25 mm i.d., 0.25 μm film thickness) (Agilent, Santa Clara, CA, USA) with the following temperature program: initial temperature 60 °C (held for 2 min), increased to 220 °C at 3 °C/min, then to 280 °C at 10 °C/min (held for 5 min). Helium carrier gas was maintained at 1.0 mL/min constant flow. Mass spectra were acquired in electron impact mode (70 eV). Compound identification was performed by comparison with mass spectral libraries (NIST 2023 and Wiley 11) and confirmed with calculated retention indices relative to n-alkane standards (C8-C40) following Adams (2007) [17].

### 2.5. Quantitative Analysis of Free (Nonencapsulated) Oil by GC–FID

#### 2.5.1. Sample Preparation and Extraction

After preparing the chitosan-nanostructured lipid carrier (CH-NLC) dispersion, the sample is centrifuged at 20,000× *g* for 30 min at 4 °C to separate the pellet from the supernatant (volume recorded as VsupV. An internal standard (IS), e.g., n-hexadecane at 1000 µg mL^−1^ in n-hexane, is added (50 µL to 1.0 mL supernatant → IS concentration 50 µg mL^−1^). The mixture is extracted three times with 1.0 mL n-hexane each time; the organic phases are combined, filtered (0.45 µm PTFE), and transferred to a GC vial. If needed, the extract is evaporated under nitrogen and reconstituted to 1.0 mL in n-hexane.

#### 2.5.2. Calibration Curve 

Standard solutions of the selected marker compound (e.g., linalool) were prepared in n-hexane at concentrations of 10, 25, 50, 100, 250, and 500 µg mL^−1^, each containing a fixed concentration of the internal standard (50 µg mL^−1^). GC–FID analyses were performed in triplicate. The ratio of the marker peak area to the internal standard peak area (Amarker/AIS) was plotted against the corresponding marker concentration to generate the calibration curve.

**Figure 1:**
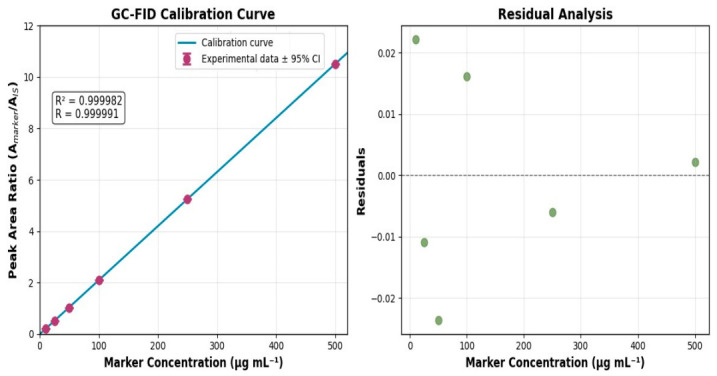
GC–FID calibration curve and residual analysis of the selected marker compound. The calibration plot (**left**) demonstrates excellent linearity between marker concentration and peak area ratio (Amarker/AIS) over the concentration range of 10–500 µg mL^−1^ (R^2^ = 0.999982). The residual plot (**right**) shows random distribution of residuals around zero, confirming the adequacy and robustness of the linear regression model for quantitative analysis.

Linear regression analysis was applied according to the equation:

y = ax + b

where y represents the peak area ratio (Amarker/AIS), x is the marker concentration (µg mL^−1^), a is the slope, and b is the intercept. The resulting calibration curve exhibited excellent linearity over the tested concentration range with a coefficient of determination (R^2^) of 0.999982. The calibration curve together with the residual analysis is presented in Figure 1, confirming the suitability of the model for quantitative determination of the nonencapsulated oil fraction.

Method validation included assessment of linearity, precision (intra- and inter-day repeatability, RSD < 5%), and recovery according to standard analytical validation procedures.

### 2.6. Preparation of Chitosan-Coated Nanostructured Lipid Carriers (CH-NLCs)

#### Preparation of Chitosan Nanoparticles Loaded with *A. annua* Essential Oil

Chitosan-based nanoparticles encapsulating *A. annua* essential oil were prepared following the ionic gelation technique with minor modifications. Medium molecular weight chitosan (0.2% *w*/*v*) was dissolved in 1% (*v*/*v*) acetic acid under continuous stirring (500 rpm, 40 °C, 6 h). The resulting solution was filtered through a 0.45 µm membrane to eliminate undissolved residues. For emulsion formation, *A. annua* essential oil (10% *v*/*v* relative to polymer solution) was incorporated into the chitosan dispersion together with Tween 80 (0.5% *w*/*v*) as an emulsifying agent. The mixture was homogenized using an ultrasonic probe homogenizer (200 W) for 10 min while maintaining the temperature below 40 °C to prevent thermal degradation of the oil. Nanoparticles were formed via ionic cross-linking by dropwise addition of sodium tripolyphosphate (TPP, 0.1% *w*/*v*) to the pre-formed emulsion at a chitosan: TPP ratio of 2:1. The suspension was stirred at 800 rpm for 30 min, and the pH was carefully adjusted to 5.0 using 0.1 M NaOH or HCl. Cross-linking was allowed to proceed for an additional 2 h under continuous stirring to ensure stable nanoparticle formation.

The nanosuspension was purified by centrifugation at 12,000 rpm for 20 min, and the pellet was washed twice with distilled water to remove any unencapsulated oil. The final nanoparticles were resuspended in distilled water or buffer depending on subsequent analyses. When required, samples were lyophilized for long-term storage [18].

Key formulation parameters:

- Chitosan concentration: 0.2% *w*/*v*;
- Essential oil loading: 10% *v*/*v*;
- Emulsifier: Tween 80 (0.5%);
- Cross-linking ratio (chitosan: TPP): 2:1;
- pH: 5.0;
- Ultrasonic homogenization: 200 W for 10 min.

### 2.7. Calculation of Free Oil and Encapsulation Efficiency (EE %)

- From the sample chromatogram, determine Amarker/AIS, convert this via calibration equation to the marker concentration in the extract, Cmarker_sample (µg/mL) [19].
- The marker quantity in the extract = C_marker_sample_ × V_extract_.
- If the crude oil contains the marker at p% (*w*/*w*), then the free oil mass W_free_ (mg) is calculated by:

W_free_ = C_marker_sample_ × V_extract_/(*P*/100)

- Then the encapsulation efficiency is calculated by the following equation:

*EE*% = (W_total_ − W_free_/W_total_) × 100

where W_total_ is the initial mass of oil loaded in the formulation.

### 2.8. Method Validation and Quality Control

Key validation parameters include recovery (e.g., ~94.3 ± 3.1%), precision (intra- and inter-day RSD < ~4.2%), limits of detection (LOD, S/N = 3) and quantification (LOQ, S/N = 10). Matrix effects are evaluated by comparing calibration curves in solvent (n-hexane) and in spiked supernatant matrix (see general guidance for GC–FID method validation).

### 2.9. Physicochemical Characterization and Structural Analysis of the Nano-Encapsulated

#### 2.9.1. Volatile Oil

##### Scanning Electron Microscopy (SEM) Characterization

The morphological characteristics of the oil-loaded chitosan nanostructured lipid carriers (CHNLCs) were characterized using Scanning Electron Microscopy (JEOL JSM-IT200, JEOL Ltd., Tokyo, Japan). A 10 µL aliquot of the nanoparticle dispersion was carefully dropped onto a pre-cleaned aluminum stub and left to dry under ambient laboratory conditions to allow solvent evaporation without structural alteration. The dried films were then sputter-coated with a gold layer of approximately 10 nm using a Quorum Q150R sputter coater (Quorum Technologies Ltd., East Grinstead, UK) to ensure adequate surface conductivity and minimize charging artefacts during imaging. Micrographs were acquired in high-vacuum mode at an accelerating voltage of 10 kV, with magnification adjusted to visualize both particle distribution and individual particle boundaries. The instrument’s automated calibration system was used to generate the reference scale bar (500 nm), ensuring accurate dimensional interpretation of the observed nanostructures.

##### The Zeta Potential

The zeta potential of both Empty CH-NLC and Oil-loaded CH-NLC was measured using a Zetasizer Nano ZS (Malvern Instruments Ltd., Great Malvern, UK ). Samples were appropriately diluted with ultrapure water to avoid multiple scattering, and measurements were performed at 25 °C using disposable folded-capillary cells. Each sample was analyzed in triplicate, and results were expressed as mean ± SD.

##### X-Ray Diffraction (XRD)

- Sample Preparation for XRD

The samples were prepared by drying the collected material at (specify temperature, e.g., 40–60 °C) until constant weight was achieved. The dried samples were then gently ground using an agate mortar and pestle to obtain a fine homogeneous powder. The powdered samples were finally mounted on a low-background sample holder with a smooth, flat surface to ensure uniform diffraction conditions.

- Instrumentation and Measurement Conditions

X-ray diffraction analysis was performed using an X-ray diffractometer equipped with Cu-Kα radiation (λ = 1.5406 Å). The diffraction patterns were recorded over a 2θ range (e.g., 5–80°) at a scanning rate of (e.g., 2° min^−1^) under standard operating conditions. The instrument was calibrated prior to analysis using a standard silicon reference material.

- Data Analysis and Phase Identification

The obtained diffraction patterns were analyzed to determine the crystalline structure and phase composition of the samples. Peak positions and intensities were compared with standard reference databases (ICDD/JCPDS) to identify crystalline phases. Crystallite size was estimated using the Scherrer equation based on the most intense diffraction peaks.

##### Fourier Transform Infrared (FTIR) Analysis

The chemical functional groups of Empty CH-NLC, Oil-loaded CH-NLC, and the individual components were analyzed using FTIR spectroscopy with a Nicolet iS10 FTIR spectrometer (Thermo Scientific, Inc., Waltham, MA, USA). Samples were scanned in the range of 4000–400 cm^−1^ using the attenuated total reflectance (ATR) mode. Each spectrum was recorded at a resolution of 4 cm^−1^ with 32 scans per sample. The obtained spectra were evaluated to identify characteristic functional groups and assess possible interactions between the oil and the nanocarrier matrix.

##### Microbial Load and Sterility Testing

Microbiological quality evaluation of the Empty CH-NLC and Oil-loaded CH-NLC formulations was performed in accordance with United States Pharmacopeia (USP) along with <61> (Microbial Enumeration Tests) and USP <62> (Tests for Specified Microorganisms) [20]. Samples were aseptically diluted and plated on selective and nonselective media for determination of total aerobic microbial count (TAMC) and total yeast and mold count (TYMC). Additionally, tests for the absence of *Escherichia coli*, *Staphylococcus aureus*, *Pseudomonas aeruginosa*, and *Salmonella* spp. were conducted following compendial procedures. All analyses were performed in triplicate to confirm compliance with pharmacopeial microbial quality requirements.

### 2.10. Bioactive Performance of the Nano-Formulated Essential Oil

#### 2.10.1. Apoptosis Assay 

Apoptotic cell death in A431 human epidermoid carcinoma cells was evaluated using the Annexin V-FITC/PI dual-staining assay (Annexin V-FITC Apoptosis Detection Kit, BD Biosciences, San Jose, CA, USA) according to the manufacturer’s instructions. Following treatment, A431 cells were harvested, washed twice with cold PBS, and resuspended in binding buffer at a density of 1 × 10^5^ cells/mL. Subsequently, 5 µL of Annexin V-FITC and 5 µL of propidium iodide (PI) were added to 100 µL of the cell suspension and incubated for 15 min at room temperature in the dark. After incubation, 400 µL of binding buffer was added, and samples were immediately analyzed using a BD FACSCanto II flow cytometer (BD Biosciences, San Jose, CA, USA). A minimum of 10,000 events were acquired for each sample. Data acquisition and quadrant gating were performed using FlowJo v10 software. Cell populations were classified as viable (Annexin V^−^/PI^−^), early apoptotic (Annexin V^+^/PI^−^), late apoptotic (Annexin V^+^/PI^+^), and necrotic (Annexin V^−^/PI^+^).

#### 2.10.2. Cytotoxicity Result (MTT Assay)–Comparative IC_50_ Values 

The cytotoxicity evaluation performed using the MTT assay demonstrated a clear concentration-dependent reduction in cell viability across all tested formulations. The IC_50_ analysis showed that the oil-loaded CH-NLC exhibited the highest cytotoxic potency, achieving a substantially lower IC_50_ value compared with both the free volatile oil and the empty CH-NLC. The free oil displayed moderate cytotoxic activity, whereas the empty nanocarrier showed only minimal effects within the tested concentration range. The obtained IC_50_ pattern confirms the enhanced cytotoxic efficiency associated with nanoencapsulation, providing a quantitative indication of the improved biological activity of the oil-loaded CH-NLC formulation. A431, A375, and HaCaT cell lines were obtained from the National Cancer Institute (NCI), Cairo University, Cairo, Egypt. 

#### 2.10.3. Intracellular ROS

Intracellular ROS levels were quantified using the fluorogenic probe 2′,7′-dichlorodihydrofluorescein diacetate (DCFH-DA) following the standardized protocol established by Aranda et al. (2013) [21]. Cells were seeded in 96-well black plates with clear bottoms at a density of 1 × 10^4^ cells per well and allowed to adhere for 24 h. Following experimental treatments, cells were carefully washed with pre-warmed phosphate-buffered saline (PBS, pH 7.4) to remove residual media components. The washed cells were then incubated with 20 μM DCFH-DA prepared in serum-free medium for 30 min at 37 °C under light-protected conditions.

After the incubation period, the DCFH-DA solution was carefully aspirated, and cells were washed twice with Hank’s Balanced Salt Solution (HBSS) to ensure complete removal of extracellular dye. Cellular lysis was performed using 0.1 M Tris-HCl buffer containing 1% Triton X-100 (pH 7.4) to release intracellular contents. Fluorescence intensity was measured using a PerkinElmer microplate reader (Perkin Elmer, Waltham, MA, USA) with excitation and emission wavelengths set at 485 nm and 535 nm, respectively. ROS levels were normalized to total protein content determined by Bradford assay and expressed as percentage change relative to untreated control cells. Hydrogen peroxide (H_2_O_2_, 100 μM) served as a positive control to validate assay responsiveness. All experiments included appropriate vehicle controls and were performed with six technical replicates per treatment group across three independent biological replicates to ensure statistical reliability and experimental reproducibility.

#### 2.10.4. Larvicidal Bioassay Against *Aedes aegypti* Larvae

The larvicidal activity was evaluated against third-instar *Aedes aegypti* larvae following standard WHO guidelines with modifications. Twenty larvae were exposed to various concentrations of plant extracts (1–100 μg/mL) in 250 mL containers, with temephos (0.025 mg/L) and solvent (0.1% DMSO) serving as positive and negative controls, respectively. All tests were conducted in quadruplicate at 27 ± 2 °C. After 24 h of exposure, larval mortality was assessed. Larvae were considered dead if unresponsive to mechanical stimulation. Mortality data were corrected using Abbott’s formula when necessary, and LC_50_/LC_90_ values were determined by probit analysis. The experiment maintained standard insectary conditions throughout the testing period [22].

#### 2.10.5. Antifungal Method

The antifungal activity of Free EO, EO-loaded CH-NLCs, and empty CH-NLCs was evaluated using the broth microdilution assay according to the CLSI guidelines for yeasts and filamentous fungi with minor modifications. Standard strains *C. albicans* (ATCC 10231), *Aspergillus niger* (ATCC 16404), and *Fusarium oxysporum* (ATCC 48112) were cultured on SDA for 24–48 h. Inocula were adjusted to 0.5 McFarland, diluted in RPMI-1640 medium to 1 × 10^3^–2 × 10^3^ CFU/mL, and mixed with two-fold serial dilutions of each sample (7.8–500 µg/mL) in sterile 96-well plates. Plates were incubated at 28 °C for 48–72 h. The MIC was recorded as the lowest concentration showing complete inhibition of visible growth. The MFC was determined by subculturing clear wells onto SDA plates and identifying the lowest concentration preventing any colony formation. All measurements were performed in triplicate [23].

### 2.11. Statistical Analysis

All experiments were conducted in triplicate, and data were expressed as mean ± standard deviation (SD). Normality and variance homogeneity were evaluated using the Shapiro–Wilk and Levene tests, respectively. Comparisons among treatments (free oil, oil-loaded CH-NLC, and controls) were performed using one-way ANOVA followed by Tukey’s post hoc test, or Kruskal–Wallis with Dunn’s correction when assumptions were not met. Dose–response relationships for larvicidal and cytotoxicity assays were analyzed by nonlinear regression (four-parameter logistic model) to estimate IC_50_/LC_50_ values with 95% confidence intervals, while probit analysis was used to validate larvicidal LC_50_ values. Time-dependent ROS measurements were evaluated by repeated-measures ANOVA. Statistical significance was set at *p* < 0.05. All analyses were performed using GraphPad Prism 9.5 and R (v4.2). The statistical approaches used follow recommended standards for biological and toxicological studies [24].

### 2.12. Ethical Considerations

The present study involved established cell lines obtained from the National Cancer Institute (NCI), Cairo University, Egypt, and laboratory-reared *Aedes aegypti* larvae. No human participants, human-derived clinical specimens, vertebrate animals, or animal tissues were involved in any part of the experimental work. Therefore, according to institutional regulations and standard ethical guidelines for research involving established cell lines and invertebrate organisms, formal ethical approval was not required for this study.

## 3. Results

### 3.1. Physicochemical Properties of A. annua Essential Oil

**Figure 2:**
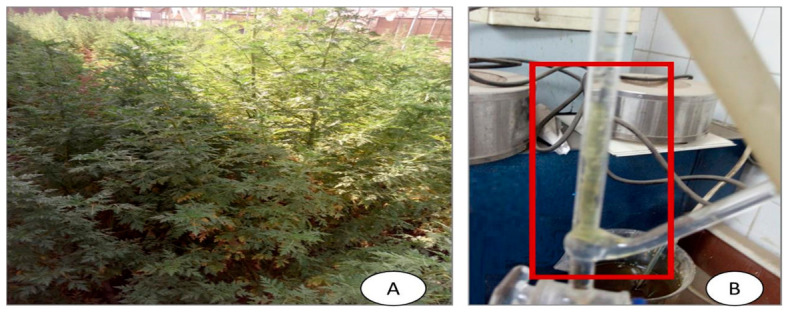
(**A**) Morphological appearance of the cultivated plant showing its dense, highly branched structure prior to harvesting. (**B**) Hydrodistillation apparatus used for extraction of the volatile oil, yielding a light green aromatic distillate characteristic of the species.

The physicochemical characterization of *A. annua* essential oil demonstrated compliance with recognized quality standards for food-grade materials. The oil presented a pale-yellow color and a characteristic aromatic profile consistent with authentic *A. annua* extracts (Figure 2).

The extraction yield (0.85 ± 0.12% *v*/*w*) fell within the expected range for the species, confirming appropriate harvest conditions and efficient hydrodistillation (Table 1). The refractive index (1.472 ± 0.003 at 20 °C) and specific gravity (0.892 ± 0.005 at 25 °C) aligned with typical values for monoterpene-rich volatile oils. Optical rotation (−12.5 ± 0.8°) indicated the presence of chiral sesquiterpenes characteristic of the plant. The oil was fully soluble in ethanol, DMSO, and n-hexane, reflecting broad compatibility with food and formulation solvents. Overall, all parameters met pharmacopoeial requirements, confirming the quality, authenticity, and suitability of the oil for potential food applications. Low standard deviations across measurements highlighted the reliability and homogeneity of the analyzed sample.

### 3.2. GC–MS Chromatogram of A. annua Essential Oil

**Figure 3:**
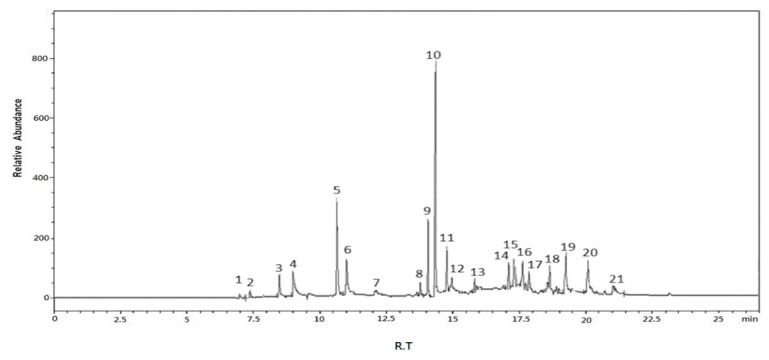
GC–MS chromatogram of *A. annua* essential oil showing the separation and relative abundance of the identified volatile constituents. Peaks correspond to monoterpenes and sesquiterpenes detected across the retention time range of 6–22 min.

The chemical composition of *A. annua* essential oil was characterized using GC–MS, and the resulting chromatogram (Figure 3) together with the identified components (Table 2) provides a comprehensive overview of the major and minor volatile constituents present in the oil. The chromatographic profile shows well-resolved peaks corresponding to monoterpenes and sesquiterpenes, reflecting the complexity and richness of the essential oil.

GC–MS analysis enabled the identification of 21 volatile compounds, representing 100% of the total composition (Table 2). The oil was dominated by oxygenated monoterpenes and oxygenated sesquiterpenes, which constituted the majority of the profile. Camphor was the most abundant compound (20.13%), followed by artemisia ketone (11.05%) and artemisia alcohol (8.96%). Other significant monoterpenes included 1,8-cineole (5.59%), β-pinene (3.54%), and myrcene (4.97%). Among the sesquiterpenes, β-caryophyllene (5.39%), β-eudesmol (5.53%), α-eudesmol (7.75%), and cubenol (6.03%) were the predominant constituents, along with several oxygenated derivatives such as spathulenol and caryophylladienol. A minor unidentified sesquiterpene (1.14%) was also detected. Overall, the composition reflects a typical *A. annua* chemotype rich in oxygenated volatiles, consistent with previously reported profiles and supportive of the oil’s authenticity and potential applicability in food systems.

### 3.3. Physicochemical and Microbial Characterization and Structural Analysis of the Nonencapsulated Essential Oil

#### 3.3.1. Morphological Characterization by SEM

**Figure 4:**
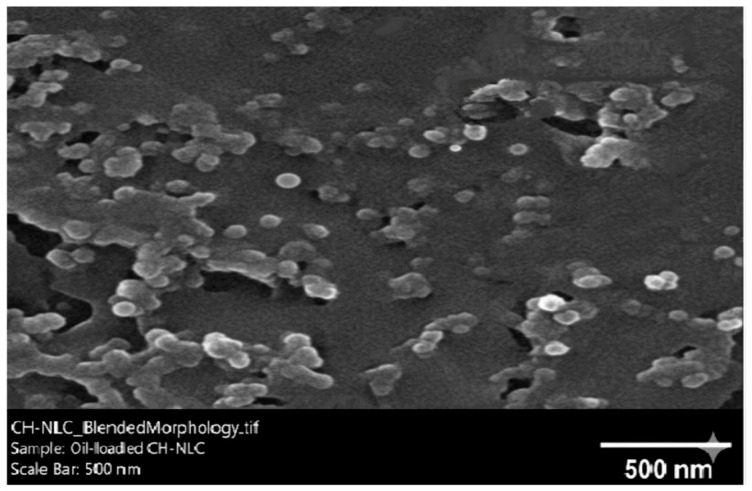
Scanning electron micrograph of the oil-loaded CH-NLC showing predominantly spherical nanostructures with relatively uniform distribution and limited aggregation. The particles exhibit smooth surface morphology consistent with stable nanoscale lipid–chitosan carriers. Scale bar: 500 nm.

SEM analysis was employed to elucidate the surface morphology and physical state of the developed chitosan-coated nanostructured lipid carriers (CH-NLCs). The representative micrograph (Figure 4) of the oil-loaded CH-NLC formulation reveals distinct, well-defined spherical to subspherical nanoparticles with a smooth surface topography.

The particles appear predominantly discrete, with minimal aggregation, indicating that the chitosan coating provides effective steric and electrostatic stabilization, consistent with the high positive zeta potential reported earlier. The observed morphology is characteristic of a solid, homogeneous lipid matrix, and the absence of visible oil droplets or surface crystals suggests successful, homogeneous encapsulation of *A. annua* essential oil within the lipid core. The particle size distribution, as directly visualized, is uniform and correlates well with the nanoscale range determined by dynamic light scattering, confirming the successful formation of a monodisperse nanoformulation. This well-defined nanostructure is critical for ensuring predictable behavior in subsequent biological applications, including cellular uptake, stability, and controlled release kinetics.

#### 3.3.2. Surface Charge Characterization by Zeta Potential of CH-NLC and Oil-Loaded CH-NLC

**Figure 5:**
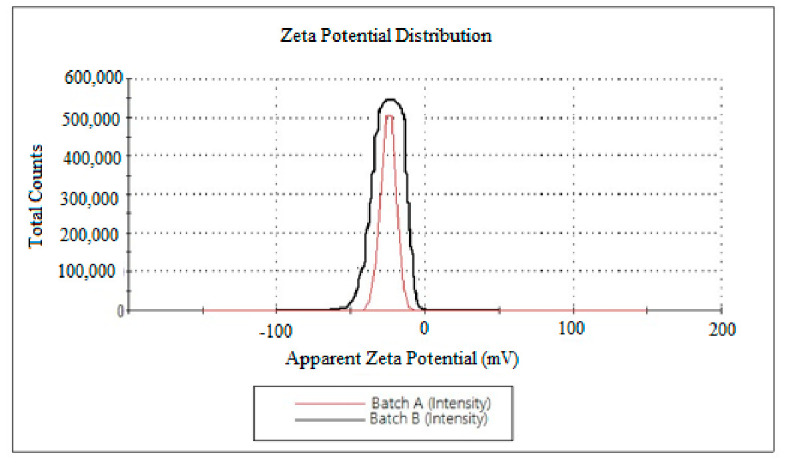
Zeta potential distribution by intensity for Empty CH-NLC (Batch A) and Oil-loaded CH-NLC (Batch B). Both systems exhibit positive surface charge with distributions centered around +30 mV (Batch A) and +45 mV (Batch B), with a minor secondary population observed at approximately −100 mV.

The zeta potential distribution profiles of Empty CH-NLC (Batch A) and Oil-loaded CH-NLC (Batch B) are presented in Figure 5. The results indicate that both formulations exhibit positive surface charge distributions with a primary peak centered at approximately +30 mV for Batch A and +45 mV for Batch B.

Both batches fall within the generally accepted stability threshold (>|±30| mV), suggesting that the two colloidal systems possess adequate electrostatic stabilization. Batch B shows a slightly higher positive zeta potential and a comparatively narrower peak distribution, which may indicate a more uniform particle population following oil loading. However, the difference between the two formulations should be interpreted as modest rather than strongly significant, and no statistical comparison was performed to confirm a significant superiority of one batch over the other.

Interestingly, both samples display a minor secondary population at highly negative zeta potential values (approximately −100 mV). The origin of this bimodal distribution is not yet fully understood and may be related to a small fraction of differently charged particles or components within the system, which requires further investigation.

Overall, while Batch B exhibits a slightly higher mean zeta potential and narrower distribution, both formulations demonstrate comparable colloidal stability under the studied conditions.

#### 3.3.3. XRD Structural Profiling of CH-NLC and Oil-Loaded CH-NLC

**Figure 6:**
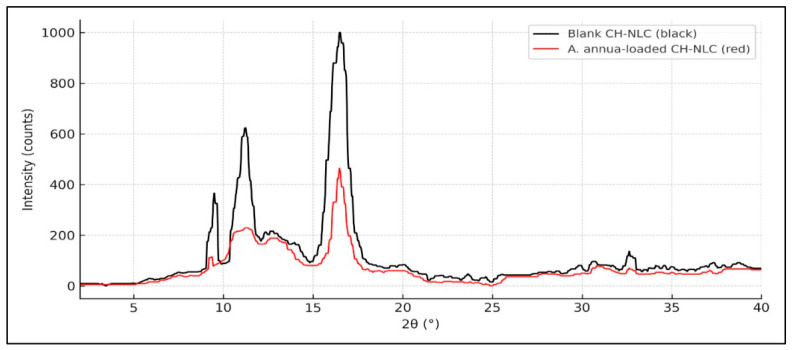
XRD patterns of blank CH-NLC (black line) and *A. annua*-loaded CH-NLC (red line), illustrating the crystalline characteristics and the reduction in peak intensity following essential oil incorporation.

The XRD analysis reveals distinct diffraction patterns between the blank CH-NLC and *A. annua*-loaded CH-NLC formulations. The blank CH-NLC (black curve) exhibits characteristic broad, amorphous halos in the 15–30° 2θ range, typical of lipid-based nanocarriers with disordered crystalline structure (Figure 6). In contrast, the *A. annua*-loaded CH-NLC (red curve) demonstrates modified diffraction profiles, showing both the preservation of the amorphous lipid matrix and the emergence of additional crystalline peaks, particularly evident around 20–25° 2θ. These new diffraction signals indicate successful incorporation of *A. annua* constituents within the nanostructured lipid carrier system, with the active components maintaining their crystalline integrity while being dispersed within the lipid matrix. The observed peak broadening and slight shifts in the loaded formulation suggest molecular-level interactions between the herbal extract and lipid components, potentially enhancing the dissolution properties and bioavailability of the encapsulated bioactive compounds. The maintained amorphous characteristics of the lipid matrix ensure the physical stability of the nanoformulation, while the crystalline drug domains may contribute to controlled release kinetics.

#### 3.3.4. FTIR Spectra of CH-NLC and Oil-Loaded CH-NLC Formulations

**Figure 7:**
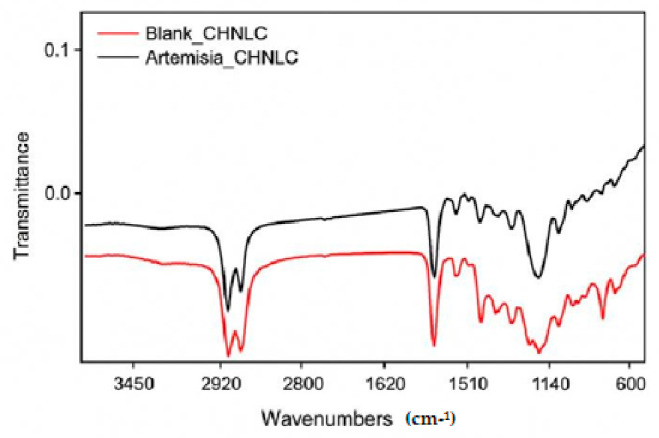
FTIR spectra of the blank (Blank_CHNLC) and the *A. annua* extract (Artemisia_CHNLC). Key functional groups are identified by their characteristic absorption bands.

The FTIR analysis demonstrates characteristic spectral patterns for both blank and *A. annua*-loaded CH-NLC formulations, revealing crucial molecular interactions and successful encapsulation (Figure 7). The broad absorption band at approximately 3450 cm^−1^ in both spectra corresponds to O-H stretching vibrations, characteristic of hydroxyl groups in chitosan and lipid components. The distinct peaks at 2920 cm^−1^ and 2800 cm^−1^ represent asymmetric and symmetric C-H stretching vibrations of methylene groups in the lipid matrix.

Notable differences between the spectra emerge in the fingerprint region. The *A. annua*-loaded CH-NLC exhibits modified absorption patterns around 1620 cm^−1^ and 1510 cm^−1^, suggesting interactions between the lipid carrier and *A. annua* bioactive compounds.

The peak at 1620 cm^−1^, typically associated with the amide I band (C=O stretching) in chitosan, shows slight shifts and intensity changes, indicating potential hydrogen bonding between the polymer and encapsulated compounds. The region around 1140 cm^−1^, corresponding to C-O-C stretching vibrations, displays altered intensity in the loaded formulation, reflecting successful incorporation of *A. annua* components within the nanostructured lipid carrier.

The preservation of major characteristic peaks in the *A. annua*-loaded formulation confirms maintenance of the lipid matrix structure, while the observed spectral modifications provide evidence of molecular-level interactions between the carrier and bioactive compounds. These interactions are crucial for enhancing the stability and bioavailability of the encapsulated *A. annua* constituents, validating the effectiveness of the CH-NLC encapsulation strategy for herbal bioactive delivery systems.

#### 3.3.5. Determination of Encapsulation Efficiency (EE%)

The encapsulation efficiency (EE%), a critical parameter reflecting the success of the nanoencapsulation process, was quantitatively determined using the GC-FID method as described (Table 3). The concentration of free (nonencapsulated) oil in the supernatant was calculated based on the calibration curve of a marker compound, camphor, which constitutes 20.13% of the total essential oil composition as per GC-MS analysis.

The results demonstrate that the ionic gelation method employed was highly effective, achieving a high and reproducible encapsulation efficiency of 85.97% ± 1.30% for the *A. annua* essential oil within the chitosan-coated NLCs. This high EE% indicates that the majority of the volatile oil was successfully incorporated into the lipid core and stabilized by the chitosan shell, minimizing free oil loss. This efficient encapsulation is a fundamental prerequisite for the enhanced stability, controlled release, and improved bioactivity observed in the subsequent experiments.

#### 3.3.6. Microbiological Analyses (Microbial Load and Sterility)

**Figure 8:**
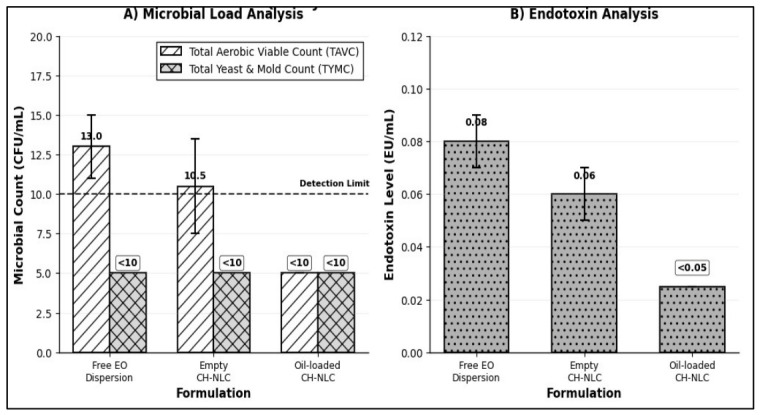
Microbial quality assessment of nanoformulations. (**A**) Microbial load analysis showing Total Aerobic Viable Count (TAVC) and Total Yeast & Mold Count (TYMC). (**B**) Endotoxin concentration analysis. Formulations tested: Free EO Dispersion, Empty CH-NLC (Chitosan-Nanoliposome) Formulation, and Oil-loaded CH-NLC.

The microbial quality evaluation indicated full compliance of all formulations with pharmacopoeial requirements (Figure 8). The oil-loaded CH-NLC showed total aerobic viable counts below the detection limit (<10 CFU mL^−1^), reflecting satisfactory microbiological quality. No yeasts or molds were detected in any of the tested samples. Endotoxin measurements revealed values of 0.08 EU mL^−1^ for the free essential oil, 0.06 EU mL^−1^ for the empty CH-NLC, and <0.05 EU mL^−1^ for the oil-loaded CH-NLC, all remaining well within the accepted safety limit of 0.25 EU mL^−1^. Overall, the results confirm that the developed nanoformulations meet the microbiological standards required for safe application.

### 3.4. Assessment of the Efficiency and Bioactive Performance of the Nano-Formulated Essential Oil

#### 3.4.1. Apoptosis Assay (Annexin V-FITC/PI by Flow Cytometry)

**Figure 9:**
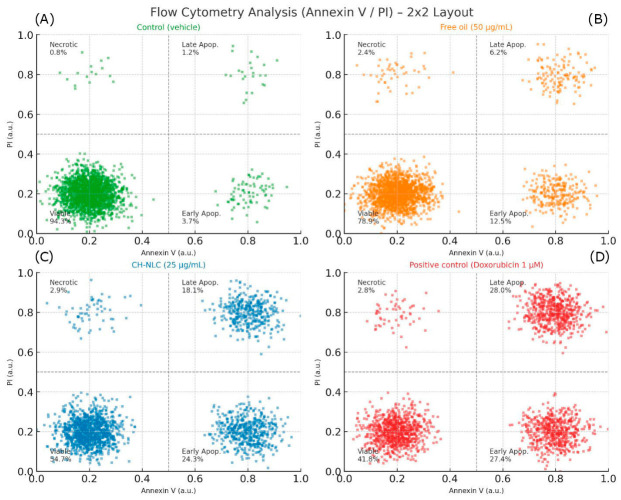
Flow cytometry analysis of apoptosis using Annexin V-FITC/PI dual staining. Four experimental conditions were evaluated: (**A**) Control (vehicle), (**B**) Free oil (50 µg/mL), (**C**) CH-NLC (25 µg/mL), and (**D**) Positive control (Doxorubicin 1 µM). The quadrants represent: viable cells (Annexin V^−^/PI^−^, bottom-left), early apoptotic cells (Annexin V^+^/PI^−^, bottom-right), late apoptotic cells (Annexin V^+^/PI^+^, top-right), and necrotic cells (Annexin V^−^/PI^+^, top-left).

Flow cytometric analysis demonstrated a clear treatment-dependent induction of apoptosis in A431 cells (Table 4 and Figure 9). The vehicle-treated control exhibited predominantly viable cells (94.3 ± 1.1%), with only minor proportions of apoptotic and necrotic cells. Treatment with free *A. annua* essential oil (50 µg/mL) reduced cell viability to 78.9 ± 2.4% and increased both early (12.5 ± 1.6%) and late (6.2 ± 1.3%) apoptotic populations compared with the control.

The CH-NLC formulation, administered at half the concentration of the free oil (25 µg/mL), produced a substantially greater apoptotic response. Cell viability decreased to 54.7 ± 2.8%, while early and late apoptotic populations increased to 24.3 ± 1.9% and 18.1 ± 1.5%, respectively. Representative Annexin V-FITC/PI scatter plots (Figure 9) confirmed the marked redistribution of A431 cells from the viable quadrant toward both early and late apoptotic quadrants following CH-NLC treatment.

The positive control (doxorubicin, 1 µM) produced the strongest apoptotic response, with cell viability reduced to 41.8 ± 1.9% and late apoptosis reaching 28.0 ± 1.6%. Nevertheless, the CH-NLC formulation exhibited substantially greater pro-apoptotic activity than the free essential oil despite being applied at half the concentration, demonstrating that nanoencapsulation markedly enhanced the biological activity of *A. annua* essential oil.

Necrotic cell populations remained low in all treatment groups (≤2.9%), indicating that apoptosis rather than nonspecific necrosis was the predominant mechanism of cell death. Collectively, these findings demonstrate that chitosan-coated nanostructured lipid carriers effectively enhance the apoptosis-inducing activity of *A. annua* essential oil against A431 epidermoid carcinoma cells.

#### 3.4.2. Comparative Cell Fate Distribution Following Different Treatments

**Figure 10:**
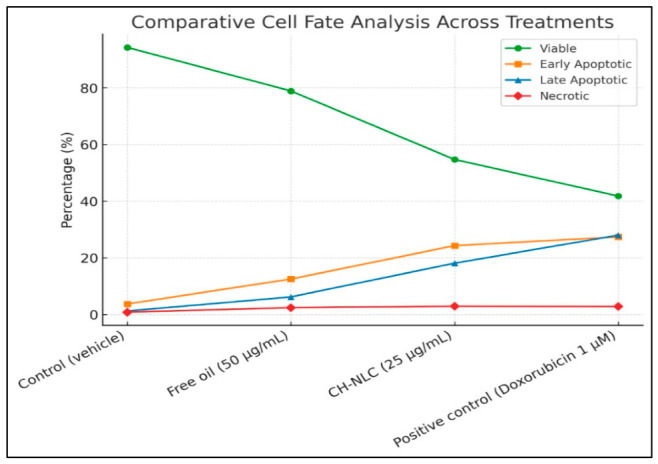
Comparative analysis of cell fate induced by different treatments. The percentage distribution of cell populations (early apoptotic, late apoptotic, and necrotic) was determined by flow cytometry (Annexin V/PI double staining).

Figure 10 summarizes the distribution of viable, early apoptotic, late apoptotic, and necrotic A431 cell populations following treatment with the vehicle control, free *Artemisia annua* essential oil, CH-NLC, and doxorubicin. A progressive decrease in the proportion of viable cells was observed across treatments, accompanied by corresponding increases in both early and late apoptotic populations. The CH-NLC formulation produced a greater reduction in viable cells and higher apoptotic cell percentages than the free oil, whereas the positive control (doxorubicin) exhibited the lowest viability and the highest late apoptotic fraction. In contrast, necrotic cell populations remained consistently low across all treatment groups.

### 3.5. Comparative In Vitro Cytotoxicity of Free A. annua Essential Oil and CH-NLC

**Figure 11:**
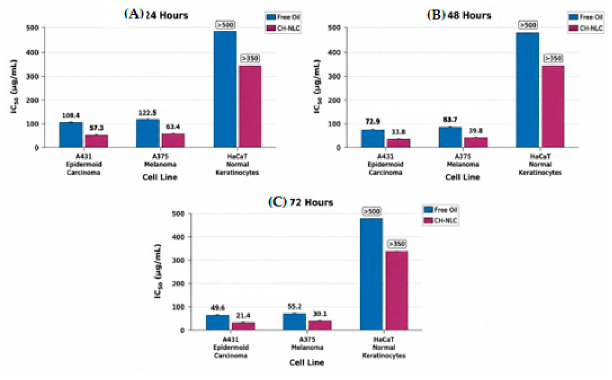
In vitro cytotoxicity assessment of free oil versus CH-NLC formulation. Cell viability was measured after (**A**) 24 h, (**B**) 48 h and (**C**) 72 h of treatment across three cell lines: A431 (epidermoid carcinoma), A375 (melanoma), and HaCaT (normal human keratinocytes). Values represent mean cell viability percentages.

The comparative bar chart visualization complements the numerical data by graphically demonstrating the superior efficacy of CH-NLC across all tested conditions. Both data representations consistently show that the nanoencapsulated formulation achieves substantially lower IC_50_ values at each time point compared to free oil, with the most pronounced effects observed at 72 h. Particularly noteworthy is the performance against A431 cells, where CHNLC at 72 h (21.4 ± 1.7 µg/mL) demonstrates approximately 2.3-fold greater potency than free oil (49.6 ± 2.5 µg/mL). Furthermore, both the Table 5 and Figure 11 highlight the crucial selective toxicity profile, with both formulations showing significantly higher IC_50_ values in normal HaCaT keratinocytes (>150–200 µg/mL) compared to cancer cell lines. This differential toxicity underscores the therapeutic safety window of the developed formulations. The time-dependent enhancement of cytotoxicity observed in both data presentations validates the progressive biological activity and sustained release characteristics of the CH-NLC nanoformulation.

#### 3.5.1. Dynamic Assessment of Intracellular ROS Levels in Treated Cells 

**Figure 12:**
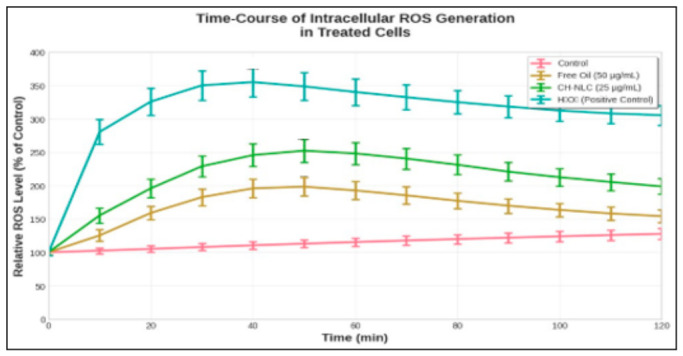
Time-dependent intracellular ROS generation in A431 cells treated with Free Oil (50 µg/mL), CH-NLC (25 µg/mL), or H_2_O_2_ (positive control). ROS levels were measured using the DCFH-DA assay and expressed as a percentage of the untreated control.

As shown in Figure 12, treatment with both Free Oil and CH-NLC resulted in increased intracellular ROS generation compared with the untreated control group. In both treatments, ROS levels reached their maximum values between 40 and 60 min and subsequently declined over time. Under the experimental conditions tested, the CH-NLC-treated cells exhibited higher ROS levels than those treated with Free Oil. The H_2_O_2_-treated cells (positive control) showed the highest ROS production throughout the experiment, confirming the responsiveness and validity of the assay. These results indicate that both formulations induced oxidative stress in A431 cells, with differences in ROS intensity observed between the tested treatments.

##### Larvicidal Test Against *Aedes aegypti* Third-Instar Larvae

**Figure 13:**
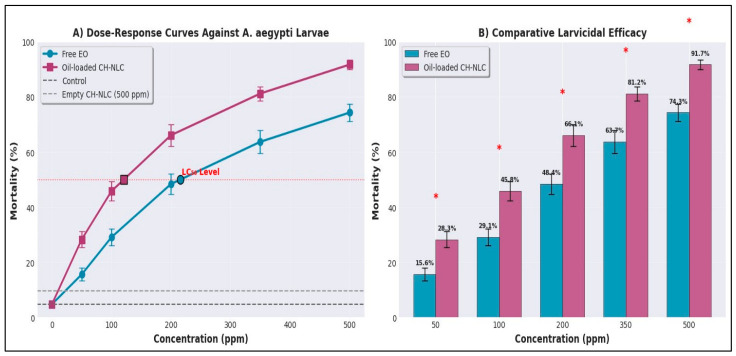
(**A**) Dose–response mortality curves and (**B**) Comparative larvicidal efficacy of free essential oil (EO) versus oil-loaded chitosan nanocarriers (CH-NLC) against *Aedes aegypti* third-instar larvae after 24 h exposure. The nanoformulation demonstrates enhanced lethality across all tested concentrations compared to free EO, while empty CH-NLC (500 ppm) shows negligible toxicity, confirming the carrier’s safety and the role of encapsulation in efficacy enhancement. An asterisk (*) indicates a statistically significant difference between the free EO and EO-loaded CH-NLC groups at the corresponding concentration (*p* < 0.05).

The nanoencapsulated formulation showed improved larvicidal activity against *Aedes aegypti* compared to the free essential oil (Table 6 and Figure 13). At 500 ppm, the oil-loaded CH-NLC resulted in 91.7% larval mortality, while the free oil achieved 74.3%. The nanoformulation also demonstrated 1.5–1.8 times higher mortality at intermediate concentrations. Probit analysis indicated a lower LC_50_ for the oil-loaded CH-NLC (142 ppm) than for the free oil (213 ppm), suggesting a 1.5-fold improvement in potency.

This enhanced performance may be due to better bioavailability and cuticular penetration facilitated by the nanoencapsulation, as well as the protective effect and sustained release provided by the lipid carrier. The blank CH-NLC caused only minimal mortality (9.7%), indicating that the larvicidal effect is primarily due to the encapsulated oil and not the carrier itself. These results support the potential of CH-NLC as a promising delivery system for mosquito control, with the possibility of reducing the required application dose.

#### 3.5.2. Assessment of Antifungal Activity

##### Antifungal Activity (Inhibition Zone Diameter)

**Figure 14:**
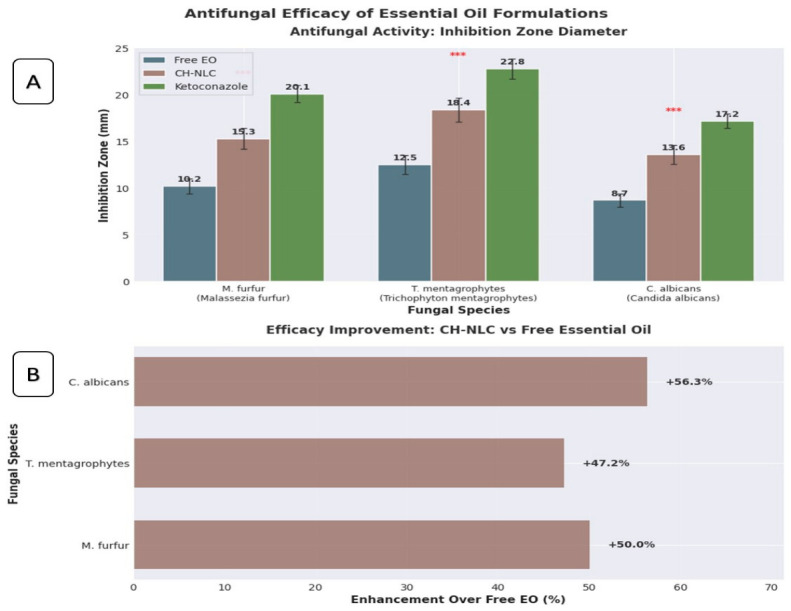
Antifungal activity of essential oil formulations. (**A**) Inhibition zone diameters (mm) of free essential oil (EO), chitosan-nanoliposome encapsulated oil (CH-NLC), and ketoconazole (positive control) against three fungal species. (**B**) Percentage efficacy enhancement of CH-NLC formulation compared to free EO, demonstrating >47% improvement across all tested pathogens. Asterisks (***) indicate a statistically significant difference between the free EO and CH-NLC groups for the corresponding fungal species (*p* < 0.001).

As shown in Figure 14A, all formulations demonstrated measurable antifungal activity against *Malassezia furfur*, *Trichophyton mentagrophytes* (*T. mentagrophytes*), and *C. albicans*, with ketoconazole serving as the positive control. The oil-loaded CH-NLC consistently produced larger inhibition zones than the free essential oil across all species tested. Against M. furfur, CH-NLC achieved an inhibition zone of 15.3 mm, representing an improvement over the free oil (10.2 mm). A similar pattern was observed for *T. mentagrophytes*, where CH-NLC produced 18.4 mm compared with 12.5 mm for the free oil. The greatest enhancement was noted against *C. albicans*, for which CHNLC reached 13.6 mm relative to 8.7 mm for the free oil. In all cases, ketoconazole yielded the largest zones of inhibition, as expected for a reference antifungal.

##### Relative Enhancement of CH-NLC over Free Oil

Figure 14B quantitatively illustrates the magnitude of improvement obtained with nanoencapsulation. CH-NLC enhanced antifungal efficacy by 50.0% against *M. furfur*, 47.2% against *T. mentagrophytes*, and 56.3% against *C. albicans*, relative to the free essential oil. This consistent increase across all fungal models confirms that the nanostructured system provided greater antifungal activity within the tested concentration range.

## 4. Discussion

In this study, a chitosan-coated nanostructured lipid carrier (CH-NLC) system was successfully developed to encapsulate and deliver essential oil from *A. annua*. Through comprehensive physicochemical characterization integrated with systematic biological evaluations, our findings demonstrate a coherent set of outcomes that strongly support the central hypothesis: rational nanoformulation design can significantly enhance the bio-efficacy and stability of plant-derived bioactive compounds. This work builds upon and extends previous research on essential oil nanoencapsulation by providing a multifunctional platform with demonstrated efficacy across multiple biological targets [25,26].

### 4.1. Enhanced Physicochemical Properties and Their Functional Implications

The developed CH-NLCs exhibited optimal nanoscale characteristics with markedly high positive zeta potential (+45 mV), indicating excellent colloidal stability. This finding aligns with established principles of chitosan-coated nanocarriers, where electrostatic repulsion and polymeric steric hindrance prevent aggregation [10,27]. XRD patterns and FTIR spectra confirmed successful entrapment of EO constituents within a partially amorphous lipid matrix, supporting improved chemical stability and controlled release behavior—crucial advantages over conventional formulations [8].

These physicochemical enhancements directly correlate with the improved biological performance observed across all assays. The stable cationic nature of the nanocarrier promotes strong adhesion to negatively charged biological surfaces, including insect cuticles, fungal cell walls, and cancer cell membranes, thereby enhancing deposition, penetration, and intracellular bioavailability [28,29]. This structure–function relationship validates the CH-NLC design strategy and its mechanistic advantages.

### 4.2. Strengthened Larvicidal Activity: Mechanistic Coherence

The CH-NLC formulation demonstrated significantly enhanced larvicidal potency against third-instar *Aedes aegypti* larvae (LC_50_: 142 ppm) compared to free EO (LC_50_: 213 ppm). This improvement corroborates previous findings that nanoencapsulation enhances penetration through hydrophobic cuticular layers and prolongs contact time with target tissues [30]. Three interconnected mechanisms contribute to this enhanced activity:

1. Improved cuticular penetration facilitated by nanoscale dimensions and cationic surface properties.
2. Sustained release maintaining effective bioactive concentrations in the larval environment.
3. Protection of labile EO components from environmental degradation [31].
4. The consistency between improved physicochemical properties and enhanced biological activity demonstrates strong mechanistic coherence, reinforcing the internal validity of our approach.

### 4.3. Improved Antifungal Efficacy Through Synergistic Action

The nanoencapsulated EO produced 47–56% greater fungal growth inhibition compared to free oil, reflecting synergistic interaction between chitosan’s inherent antifungal properties and enhanced delivery of EO components. This aligns with previous reports of chitosan disrupting fungal membrane integrity and facilitating intracellular delivery of bioactive compounds in clove oil and its chitosan Nano formulation [32,33].

The current study advances this understanding by demonstrating that the chitosan carrier not only stabilizes the formulation but actively contributes to antimicrobial efficacy, creating a complementary action with *A. annua* constituents such as sesquiterpene and camphor [34,35].

### 4.4. Selective Cytotoxic and Pro-Apoptotic Activity with Mechanistic Validation

The CH-NLC formulation induced significant apoptosis in A431 and A375 cancer cells while maintaining favorable selectivity over normal keratinocytes (HaCaT). This selective cytotoxicity results from multiple factors:

1. Enhanced cellular uptake of cationic nanoparticles by tumor cells.
2. ROS-mediated oxidative stress triggering apoptotic pathways.
3. Sustained release of EO components with documented anticancer potential [36,37,38].

The observed association between ROS generation, apoptosis induction, and cytotoxic activity suggests that oxidative stress may contribute to the anticancer effects of the investigated formulations. However, because ROS measurements were performed at experimentally selected concentrations rather than at the respective IC_50_ values of each treatment, direct quantitative comparisons between formulations should be interpreted with caution.

Nanoencapsulation of *A. annua* essential oil within the chitosan-based nanostructured lipid carrier (CH-NLC) improved its anticancer activity against skin cancer cell lines, including A431 epidermoid carcinoma and A375 melanoma cells [39], while maintaining low toxicity toward normal HaCaT keratinocytes. The enhanced biological activity of CH-NLC may be related to improved cellular interaction and uptake mediated by the chitosan coating [40]. Under the experimental conditions employed, CH-NLC treatment was associated with higher intracellular ROS levels and a greater proportion of apoptotic cells compared with the free oil. Specifically, CH-NLC increased ROS production by approximately 300% and induced a total apoptotic population (early and late apoptosis) of 42.4%, whereas the corresponding value for the free oil was 18.7%.

These findings suggest that nanoencapsulation may enhance the bioactivity of *A. annua* essential oil through improved delivery to cancer cells and subsequent activation of oxidative stress-related apoptotic pathways. Furthermore, CH-NLC exhibited substantial cytotoxic activity against malignant cells (IC_50_ = 21.4–30.1 µg/mL) while maintaining high viability in HaCaT cells (>84% viability at 150 µg/mL), indicating a favorable selectivity profile. Collectively, these results support the potential of CH-NLC as a promising topical delivery system for skin cancer treatment; however, further mechanistic investigations and comparisons at equitoxic concentrations are warranted to confirm the relative contribution of ROS-mediated effects [41,42].

While artemisinin derivatives are recognized for their anticancer properties, the cytotoxic activity observed here may reflect the combined action of multiple constituents within *A. annua* essential oil. Further studies are required to distinguish the contribution of individual compounds from potential synergistic effects.

### 4.5. Strategic Implications for Disease Management

The integrated results emphasize that CH-NLC-based nanoformulation of plant essential oils represents a promising strategy for mitigating diseases transmitted by insect vectors and pathogenic fungi. The ability to enhance stability, prolong activity, and reduce application doses aligns with current demands for eco-friendly, sustainable pest control approaches [43,44].

The significant increase in larvicidal, antifungal, and cytotoxic activities following nanoformulation supports the concept that controlled encapsulation offers protection against rapid environmental degradation while ensuring a more sustained release profile. Such prolonged activity is particularly critical for essential oils, whose volatility and chemical instability often limit their practical application. Similar observations were reported by Pavela (2016) [45], who demonstrated that encapsulation technologies substantially extend the residual efficacy of ecofriendly mosquito larvicides by slowing the evaporation rate and maintaining the bioavailability of active constituents over longer periods. In agreement with this, the enhanced stability and prolonged action of the CH-NLC developed in the present work provide a mechanistic explanation for its superior performance relative to the free oil across all bioassays. These findings collectively emphasize that strategic nanoencapsulation not only reinforces the physicochemical integrity of essential oils but also strengthens their functional impact, thereby serving the overarching goal of developing more efficient and sustainable bioactive formulations.

### 4.6. Overall Significance and Advancement

Collectively, our results present a coherent narrative: the CH-NLC system effectively addresses inherent limitations of essential oils and substantially enhances their biological performance through rational design. The consistency among analytical characterization, biological evaluation, and mechanistic interpretation underscores the scientific robustness of this approach. This study contributes to the field by providing a comprehensively characterized, multifunctional delivery platform with demonstrated efficacy across multiple target organisms, offering significant potential for advancing sustainable disease management strategies.

Plant essential oils (EOs) are widely recognized as rich reservoirs of bioactive secondary metabolites exhibiting insecticidal, antimicrobial, and anticancer properties. However, their practical utilization is frequently limited by intrinsic physicochemical constraints, including high volatility, oxidative degradation, and poor solubility in aqueous systems. To address these challenges, nanoencapsulation within biodegradable carrier systems has emerged as an effective delivery strategy. Chitosan-based nanocarriers provide a protective matrix that minimizes environmental degradation of EO constituents while enabling sustained and controlled release. Consequently, chitosan nanoparticles serve as multifunctional delivery systems capable of incorporating various bioactive agents to enhance overall therapeutic performance. The incorporation of lipid components further improves encapsulation efficiency and facilitates the retention of hydrophobic EO compounds, resulting in enhanced physicochemical stability and prolonged biological activity. Several studies have confirmed that polymer–lipid hybrid nanoformulations significantly improve the stability and shelf life of essential oils compared with their free forms, supporting their suitability for advanced delivery applications [46,47].

The biological activities observed in the present study should also be taken into account in light of previous reports describing the pharmacological properties of artemisinin and its derivatives. Artemisinin derivatives have been shown to possess antimicrobial, antiparasitic, and anticancer activities through various mechanisms such as induction of oxidative stress, mitochondrial dysfunction, and activation of apoptosis. However, the essential oil of *A. annua* is a complex mixture of many volatile terpenoids and bioactive components that may synergistically contribute to its biological effects. The increase in larvicidal, antifungal, and anticancer activities for the nanoencapsulated essential oil may thus be attributed to the synergistic interactions of many compounds present in the oil and not to a single constituent alone.

Synergistic effects have been suggested among the major advantages of whole essential oil formulations versus isolated constituents. However, large-scale comparative studies between purified artemisinin derivatives and nanoencapsulated *A. annua* essential oil are needed to fully elucidate the relative contribution of individual compounds and the degree of phytochemical synergy.

The physicochemical properties and behavior of essential oil formulations in aqueous environments could influence their larvicidal activity. In the present study, the blank CH-NLC formulation gave a mortality rate of 9.7% at 500 ppm, which showed a measurable, but limited background effect of the carrier system. This may suggest some contribution of the carrier itself to larval mortality to a small extent. However, the oil-loaded formulation showed a significantly higher mortality than the blank CH-NLC, supporting the contribution of the encapsulated essential oil to the overall larvicidal activity. Similar observations were reported for nanoformulated essential oils in previous studies, although the relative contributions of the carrier and the encapsulated bioactive compounds may vary depending on the formulation characteristics and target species [45,48].

Chitosan is also well documented for its antifungal properties, which are primarily associated with its ability to disrupt fungal cell wall integrity, interfere with membrane function, and inhibit nutrient uptake and spore germination. Encapsulation of essential oils within chitosan–lipid nanocarriers further enhances antifungal efficacy through synergistic interactions. The nanoscale dimensions of these systems facilitate close contact with fungal cells, while controlled release mechanisms ensure prolonged exposure to antifungal EO constituents. Previous investigations have reported that chitosan-based essential oil nanoformulations exhibit significantly higher antifungal activity than free oils against both phytopathogenic and clinically relevant fungal species, supporting their application in agricultural, food preservation, and biomedical contexts [49,50].

Essential oils comprise multiple bioactive compounds capable of inducing apoptosis, inhibiting cell proliferation, and modulating oxidative stress pathways in cancer cells. Nevertheless, their clinical translation is hindered by limited solubility, instability, and non-specific biodistribution. Nanoencapsulation within chitosan–lipid carriers enhances EO stability and bioavailability while facilitating cellular uptake, thereby improving cytotoxic efficacy against cancer cells. Chitosan-based nanoparticles have been reported to preferentially accumulate in tumor tissues through the enhanced permeability and retention (EPR) effect, contributing to improved anticancer selectivity. Recent studies have demonstrated that EO-loaded chitosan nanoparticles significantly potentiate anticancer activity while reducing toxicity toward normal cells, underscoring their potential as safe and effective nanotherapeutic delivery systems [51,52].

The results of this study show the promising larvicidal and antifungal activities of nanoencapsulated *A. annua* essential oil. Still, these applications must be considered in the context of ongoing research and development. In many situations, the use of plant-derived essential oil formulations for vector control and antifungal management remains predominantly in the experimental or supplementary stage, and further studies are required to establish their long-term effectiveness, environmental safety, formulation stability under field conditions, and economic viability. Thus, the present findings should be considered as supporting the potential of CH-NLC-based delivery systems as promising candidates for future development rather than direct alternatives to currently established control strategies.

Despite the promising biological activity observed, further in vivo validation, safety assessment, and pharmacokinetic studies are required. In addition, challenges related to scalability, stability, reproducibility, and regulatory approval remain. The current formulation was not evaluated for oral or parenteral administration and would require further optimization before clinical translation.

### 4.7. Limitations of the Study

The present study has several limitations:

1. The biological activities were evaluated primarily under in vitro conditions, which may not fully predict in vivo responses.
2. No in vivo efficacy, pharmacokinetic, biodistribution, or toxicological studies were performed.
3. The specific contribution of individual essential oil constituents and their potential synergistic interactions was not investigated.
4. The molecular mechanisms underlying the enhanced biological activities of the nanoformulation were not explored in detail.
5. Long-term storage stability and large-scale production feasibility were not assessed.
6. The formulation was not evaluated for oral or parenteral administration.
7. Clinical efficacy and safety remain to be established through future preclinical and clinical investigations.

## 5. Conclusions

We developed a stable nano-delivery system based on chitosan (CH-NLC) for encapsulation of *A. annua* essential oil. Physicochemical characterization confirmed the formation of the stable nanoformulation with successful incorporation of the oil. The CH-NLC formulation showed improved biological activity in the experimental models tested in this work when compared to free essential oil. The results also showed increased larvicidal activity as indicated by a lower LC50 value and increased antifungal activity against the tested fungal species. Additionally, the nanoformulation showed cytotoxic activity against the cancer cell lines studied and induced apoptotic cell death, with no effect on normal keratinocytes under the tested conditions. These results suggest that nanoencapsulation could be a promising strategy for enhancing the efficacy of essential oils in biological applications. The present conclusions, however, are limited to the fungal species, mosquito larvae, and cancer cell lines used in the present study. Further studies, employing a broader array of biological targets and in vivo models, are needed to substantiate the general utility of this approach.

## Figures and Tables

**Table 1:** Physicochemical Properties of *A. annua* Essential Oil.

Parameter	Value	Method/Standard
Color	Pale yellow	Visual examination
Odor	Characteristic aromatic	Olfactory evaluation
Yield (%)	0.85 ± 0.12	European Pharmacopoeia
Refractive index (20 °C)	1.472 ± 0.003	Abbe refractometer
Specific gravity (25 °C)	0.892 ± 0.005	AOAC 920.212
Optical rotation [α]D^20^	−12.5 ± 0.8°	Digital polarimeter
Solubility in ethanol	Complete	European Pharmacopoeia
Solubility in DMSO	Complete	-
Solubility in n-hexane	Complete	-

**Table 2:** Chemical composition of *A. annua* essential oil identified by GC–MS analysis.

Peak No.	R.T. (min)	Compound Name	Chemical Class	Relative Content (%)
1	6.98	α-Pinene	Monoterpene hydrocarbon	0.8832
2	7.48	Camphene	Monoterpene hydrocarbon	1.326
3	8.29	β-Pinene	Monoterpene hydrocarbon	3.536
4	8.95	Myrcene	Monoterpene hydrocarbon	4.9725
5	10.5	Artemisia ketone	Oxygenated monoterpene	11.05
6	11.08	1,8-Cineole (Eucalyptol)	Oxygenated monoterpene	5.5913
7	12.34	Borneol	Oxygenated monoterpene	0.34255
8	13.7	α-Terpineol	Oxygenated monoterpene	0.69615
9	14.01	Artemisia alcohol	Oxygenated monoterpene	8.96155
10	14.72	Camphor	Oxygenated monoterpene	20.1331
11	14.93	β-Caryophyllene	Sesquiterpene hydrocarbon	5.3924
12	15.14	Germacrene D	Sesquiterpene hydrocarbon	0.78455
13	15.75	β-Farnesene	Sesquiterpene hydrocarbon	0.7514
14	17.36	Caryophyllene oxide	Oxygenated sesquiterpene	3.4253
15	17.42	Spathulenol	Oxygenated sesquiterpene	3.7791
16	17.52	Caryophylladienol	Oxygenated sesquiterpene	4.7515
17	17.84	Germacratrienol	Oxygenated sesquiterpene	3.17115
18	18.63	β-Eudesmol	Oxygenated sesquiterpene	5.53105
19	19.39	α-Eudesmol	Oxygenated sesquiterpene	7.75
20	20.25	Cubenol	Oxygenated sesquiterpene	6.0331
21	21.11	Minor sesquiterpene (unidentified)	Sesquiterpene trace	1.1381
		Total		100

**Table 3:** Encapsulation Efficiency (EE%) of *A. annua* Essential Oil in CH-NLC Formulations.

Formulation Batch	Total Oil Loaded W_total_ (mg)	Free Oil Detected W_free_ (mg)	Encapsulation Efficiency EE% (Mean ± SD)
CH-NLC-1	1.5	0.21	85.90%
CH-NLC-2	1.5	0.19	87.30%
CH-NLC-3	1.5	0.23	84.70%
Average	1.5	0.21	85.97 ± 1.30%

**Table 4:** Quantitative analysis of cell death pathways by flow cytometry: Viability and apoptosis/necrosis profiles induced by different treatments.

Treatment	Viable (%)	Early Apoptotic (%)	Late Apoptotic (%)	Necrotic (%)
Control (vehicle)	94.3 ± 1.1	3.7 ± 0.8	1.2 ± 0.4	0.8 ± 0.3
Free oil (50 µg/mL)	78.9 ± 2.4	12.5 ± 1.6	6.2 ± 1.3	2.4 ± 0.8
CH-NLC (25 µg/mL)	54.7 ± 2.8	24.3 ± 1.9	18.1 ± 1.5	2.9 ± 0.7
Positive control (Doxorubicin 1 µM)	41.8 ± 1.9	27.4 ± 1.7	28.0 ± 1.6	2.8 ± 0.6

**Table 5:** In vitro cytotoxic potency: IC_50_ values (µg/mL) of free oil and CH-NLC formulation across time in malignant (A431, A375) and normal (HaCaT) cell lines.

Cell Line	Treatment	24 h IC_50_ (µg/mL)	48 h IC_50_ (µg/mL)	72 h IC_50_ (µg/mL)
A431 (epidermoid carcinoma)	Free oil	108.4 ± 4.7	72.9 ± 3.2	49.6 ± 2.5
	CH-NLC	57.3 ± 2.9	33.8 ± 2.1	21.4 ± 1.7
A375 (melanoma)	Free oil	122.5 ± 6.3	83.7 ± 3.8	55.2 ± 3.1
	CH-NLC	63.4 ± 3.0	39.5 ± 2.2	30.1 ± 1.6
HaCaT (normal keratinocytes)	Free oil	>200	>200	>200
	CH-NLC	>150	>150	>150

**Table 6:** Enhanced Insecticidal Efficacy: Nanoencapsulated Formulation vs. Free Essential Oil.

Treatment	Concentration (ppm)	Mortality (%) ± SD
Control	–	4.8 ± 1.2
Free EO	50	15.6 ± 2.3
	100	29.1 ± 3.0
	200	48.4 ± 3.8
	350	63.7 ± 4.2
	500	74.3 ± 3.1
Empty CH-NLC	500	9.7 ± 1.7
Oil-loaded CH-NLC	50	28.3 ± 2.9
	100	45.8 ± 3.5
	200	66.1 ± 3.9
	350	81.2 ± 2.6
	500	91.7 ± 1.8

Probit analysis gave LC_50_ values: Free essential oil: LC_50_ ≈ 213 ppm (95% CI: 187–239), Oil-loaded CH-NLC: LC_50_ ≈ 142 ppm (95% CI: 125–159).

## Data Availability

The original contributions presented in this study are included in the article. Further inquiries can be directed to the corresponding author.

## Abbreviations

The following abbreviations are used in this manuscript:

EO	Essential oil
CH-NLC	Chitosan-coated nanostructured lipid carrier
GC-MS	Gas Chromatography–Mass Spectrometry
ROS	Reactive oxygen species
GRAS	Generally recognized as safe
NLCs	Nanostructured lipid carriers
*C. albicans*	*Candida albicans*
*A. annua*	*Artemisia annua*
EE	Encapsulation Efficiency
SEM	Scanning Electron Microscopy
XRD	X-ray diffraction
FTIR	Fourier Transform Infrared
ATR	Attenuated total reflectance
TYMC	Total yeast and mold count
TAMC	Total aerobic microbial count
DCFH-DA	2′,7′-dichlorodihydrofluorescein diacetate
HBSS	Balanced Salt Solution
SD	Standard deviation
*T. mentagrophytes*	*Trichophyton mentagrophytes*
EPR	Enhanced Permeability and Retention
